# The association between chronic rhinosinusitis and the risk of dementia: a longitudinal study

**DOI:** 10.3389/fnagi.2025.1609790

**Published:** 2025-10-06

**Authors:** Xinyu Zhang, Zhongmin Yin, Jia Luo, Runsheng Yang, Weijing Wang, Dongfeng Zhang

**Affiliations:** ^1^Department of Epidemiology and Health Statistics, School of Public Health, Qingdao University, Qingdao, China; ^2^Outpatient and Emergency Department, The Affiliated Hospital of Qingdao University, Qingdao, China

**Keywords:** chronic rhinosinusitis, dementia, mediation effect, epidemiology, longitudinal study

## Abstract

**Background:**

Chronic inflammation status could increase the risk of dementia, and chronic rhinosinusitis (CRS) could cause chronic inflammation status. Therefore, CRS may be associated with dementia. The aim of our study was to investigate the association between CRS and the risk of dementia in the UK Biobank (UKB) cohort.

**Materials and methods:**

A total of 3,64,945 participants were included in this cohort study. CRS information was obtained from the first occurrence date of CRS (Field 131,468) at baseline. A Cox regression model and mediation analysis were performed to measure the association between CRS and dementia.

**Results:**

Chronic rhinosinusitis was significantly associated with an increased risk of Alzheimer’s disease (AD) (hazard ratio [HR]: 1.33, 95% CI: 1.04–1.71) but was not associated with the risk of all-cause dementia (hazard ratio [HR]: 1.04, 95% CI: 0.86–1.26) or vascular dementia (VD) (hazard ratio [HR]: 0.65, 95% CI: 0.40–1.07). The male participants, individuals with hypertension, former smokers, participants with less than a college-level education, and participants with a medium-level polygenic risk score for Alzheimer’s disease (PRS-AD) were more susceptible to AD. Mediation analysis using the comprehensive inflammatory index showed that the systemic immune-inflammation index (SII) could explain 0.0042 of this association.

**Conclusion:**

Chronic rhinosinusitis may be associated with a higher risk of AD, and the association was mediated, in a very small part, by the SII.

## 1 Introduction

Dementia is a common neurodegenerative disease among older adults and includes different sub-phenotypes, such as Alzheimer’s disease (AD), vascular dementia (VD), and frontotemporal dementia. AD and VD account for 60% ~ 80 and 15% ~ 20% of all dementia cases (Wolters and Arfan Ikram, 2019; Erkkinen et al., 2018; Raz et al., 2016). According to the World Health Organization (WHO) reports in 2023, the number of people living with dementia worldwide had reached 55 million, with nearly 10 million new cases appearing annually. Dementia causes approximately 1.3 trillion US dollars in economic losses to the global economy each year (Dementia, 2025). In addition, as a non-communicable chronic disease (NCD), it is essential to investigate the potential risk factors and implement corresponding measures to prevent and control it. Some studies have found that various factors could increase the risk of dementia, such as aging (Jin et al., 2024), less activity (Raichlen et al., 2023), education (Lövdén et al., 2020), social participation (Sommerlad et al., 2023), and smoking status (Livingston et al., 2024; Durazzo et al., 2014). Furthermore, other NCDs could also increase the risk of dementia, for example, hypertension (Barnes and Yaffe, 2011), diabetes (Biessels and Despa, 2018), and obesity (Tang et al., 2021).

Chronic rhinosinusitis (CRS) is a disease with a high recurrence rate (Hamilos, 2011; Xie et al., 2023). Patients with CRS typically experience insomnia, facial pain/pressure, anosmia, and persistent (often ≥12 weeks) inflammation of the nasal or sinus mucosa (Hamilos, 2011). A mechanistic study revealed that nasal cavity inflammation could propagate through the olfactory bulb and olfactory neural system, potentially serving as a link between CRS and dementia (Harrass et al., 2021). Several studies’ results have shown that inflammation status may be associated with the risk of dementia (Zhang et al., 2022; Luo et al., 2022; Patani et al., 2023). In addition, some cross-sectional studies have shown that CRS might be a risk factor for AD or cognitive dysfunction (Jung et al., 2021; Chung et al., 2015). However, the results of a longitudinal study and a nested case–control study showed that CRS was not associated with dementia or the subtypes of dementia, such as AD and Parkinson’s disease (Wee et al., 2020). Therefore, the association between CRS and the risk of dementia warrants further attention.

Given the inconsistency of the above research results, the lack of research exploring the mediating role of inflammation in CRS and dementia, and the lack of large-scale cohort studies in this field, we conducted this cohort study to evaluate the association between CRS and the risk of dementia using the UK Biobank (UKB) and explore whether inflammatory factors mediate the association between CRS and dementia. Investigating the association among CRS, dementia, and inflammation can help understand the relationship between different organs of the body and disease processes.

## 2 Materials and methods

### 2.1 Study population and design

**Figure 1:**
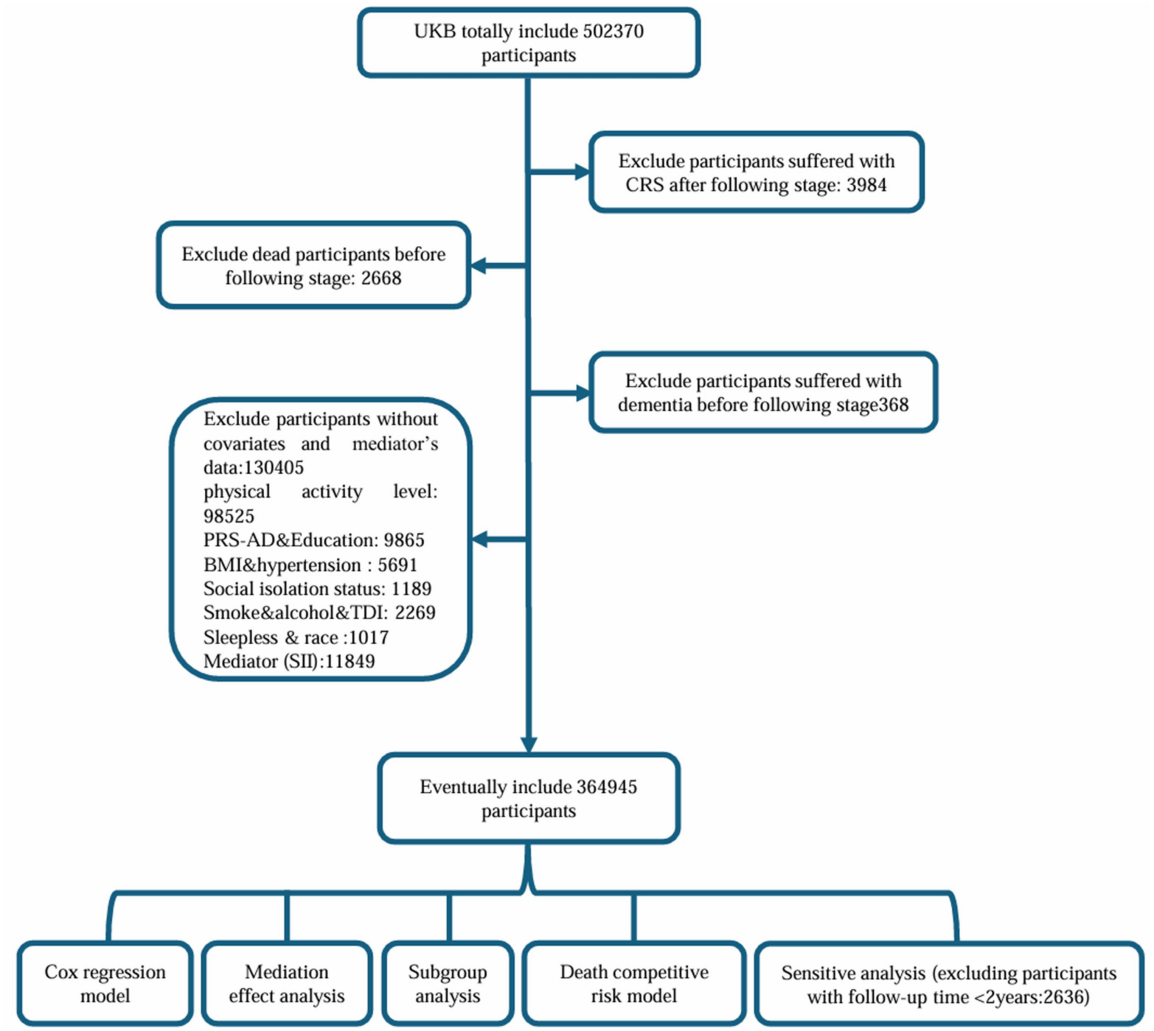
Flowchart of the study.

The UK Biobank (UKB) is a prospective cohort study that recruited more than 5,00,000 participants from 22 assessment centers across the UK from 2006 to 2010. The baseline data of the participants were obtained from the UKB, including sex, age, and ethnicity. The follow-up period was from 1st January 2011 to 1st January 2023. The time from the start of the follow-up to the incidence of dementia or death was used as the survival time variable in this analysis. We excluded patients who developed CRS after the start of the follow-up (*N* = 3,984), those who died before the start of the follow-up (*N* = 2,668), those with dementia at baseline (*N* = 368), and those without covariates data (*N* = 130,405). The flowchart of the study design is presented in Figure 1. Ethical approval was obtained from the National Information Governance Board for Health and Social Care and the National Health Service Northwest Multi-Center Research Ethics Committee. All participants provided informed consent via electronic signature prior to enrollment.

### 2.2 Chronic rhinosinusitis diagnosis

We used Field 131,468 and Field 41,270 to identify CRS, which included chronic maxillary sinusitis, chronic frontal sinusitis, chronic ethmoidal sinusitis, chronic sphenoidal sinusitis, chronic pansinusitis, other chronic sinusitis, and unspecified chronic sinusitis. These variables were used to diagnose patients with CRS according to the International Statistical Classification of Diseases and Related Health Problems, 10th Revision (ICD-10) code J32, and to determine the first occurrence date of CRS. Ultimately, we included 7,176 patients with CRS.

### 2.3 Dementia diagnosis

We divided dementia into three categories: all-cause dementia, AD, and VD. The diagnosis of dementia and its classifications were conducted according to the International Classification of Diseases, Ninth Revision (ICD-09) codes and ICD-10 codes. We used the variables—algorithmically defined dementia outcomes and ICD-10 codes—to determine the date of dementia diagnosis. We included 5,329 dementia patients, including 2,538 AD cases and 1,232 VD cases. Other disease types were not studied due to insufficient case numbers. The details of dementia diagnoses and its sub-phenotypes are provided in Supplementary materials 1, 2.

### 2.4 Covariates

Relevant covariates were collected at baseline. Given that dementia is a chronic NCD and based on prior knowledge from previous studies, many factors may influence its occurrence. Therefore, we included the following covariates: demographic factors such as age, sex, ethnicity, education, and the Townsend deprivation index (TDI); lifestyle factors including alcohol consumption status, smoking status, body mass index (BMI), and physical activity level (Cassidy et al., 2016); and disease-related factors, adjusting for hypertension, diabetes, stroke, and cancer. In addition, we included social isolation status, sleeplessness, and a standardized polygenic risk score for Alzheimer’s disease (PRS-AD) as covariates. People with social isolation are more prone to dementia, and older adults are more likely to experience social isolation, which may affect the occurrence of dementia (Ren et al., 2023). Regarding sleeplessness, patients with CRS usually experience sleep disturbances, which can reduce quality of life (Tarasidis et al., 2015) and impair cognitive performance (Wardle-Pinkston et al., 2019). These effects may play a critical role in the long-term development of dementia. In addition, dementia is a polygenic hereditary disease. Hence, we adjusted for the PRS-AD to control for the genetic factors associated with dementia. The details of the covariates are provided in Supplementary material 3.

### 2.5 Mediators

The UKB used Beckman Coulter LH750 instruments to analyze blood samples from 500,000 participants, which were collected in 4 mL EDTA vacutainers. The LH750 hematology analyzer is a quantitative, automated hematology analyzer and leukocyte differential counter. We selected inflammation indexes, such as the systemic immune-inflammation index (SII, neutrophils*platelets/lymphocytes), the neutrophil-to-lymphocyte ratio (NLR, neutrophils/lymphocytes), and the platelet-to-lymphocyte ratio (PLR, platelets/lymphocytes). To minimize the effect of extreme values and achieve a more normal or symmetrical data distribution, we used the 1st and 99th percentiles as convergence values and log-converted the data. The histogram of the log-converted SII is shown in Supplementary material 4.

### 2.6 Statistical analysis

Baseline characteristics were summarized according to CRS status: mean (standard deviation [SD]) for continuous variables with a normal or symmetric distribution, median (interquartile Range [IQR]) for continuous variables with a non-normal distribution, and number (percentage) for categorical variables. We used the chi-squared test for categorical variables, the *t*-test for continuous variables with a normal distribution, and the Mann–Whitney *U* test for continuous variables with a non-normal distribution.

We used a Cox proportional hazards regression model to evaluate the association between CRS and the risk of different dementia phenotypes. We tested the proportional hazards assumption of the Cox regression model using the Schoenfeld test, and the results of the Schoenfeld test did not violate the proportional hazards assumption (Supplementary material 5). The results were reported as hazard ratios (HRs) with 95% confidence intervals. The timescale was defined as the follow-up time (in years) from the beginning of the follow-up to the date of dementia diagnosis and/or death. We conducted three models. Model 1 was a crude model without any covariates. In Model 2, we adjusted for age, sex, ethnicity, BMI, TDI, and PRS-AD. Based on Model 2, Model 3 included additional adjustments for education level, alcohol consumption status, smoking status, physical activity level, social isolation status, sleeplessness, hypertension, diabetes, stroke, and cancer. In addition, we performed Kaplan–Meier (K–M) survival analysis for the cumulative incidence of dementia and different sub-phenotypes.

In the mediation analysis, we used the Process package in SPSS to evaluate the mediating role of inflammatory factors. This analysis provided estimates of the direct effect, indirect effect, total effect, and mediation proportion of inflammatory factors. The direct effect was the impact of CRS on dementia. The indirect effect was calculated as a*b, where “a” represented the estimated value of the impact of CRS on the SII and “b” represented the estimated value of the impact of the SII on the outcome. The total effect was calculated as direct effect + indirect effect, and the mediating proportion was calculated as indirect effect/total effect * 100% (Tönnies et al., 2023). The mediation analysis used 1,000 bootstrapping simulations.

To further explore whether the effects of CRS on dementia varied across individual characteristics, subgroup analyses were performed by sex, hypertension, smoking status, education level, and PRS-AD. In addition, three sensitivity analyses were performed to test the robustness of the association between CRS and dementia. First, we performed a competitive risk model, and the competitive outcome was death. Second, we excluded participants with ≤2 years of follow-up because dementia is an NCD that usually has a long preclinical stage. Finally, we excluded self-reported CRS cases and performed Cox regression, as self-reported CRS cases may be inaccurate. All statistical analyses were conducted using R 4.4.1 and SPSS 24.0. Statistical significance was defined as a two-sided *p*-value of <0.05.

## 3 Results

### 3.1 Participants’ baseline characteristics

Overall, the median age of the participants at baseline was 60 (13) years, with 173,598 (47.6%) male participants, and the median follow-up time was 12 years. Compared to the non-CRS group, the CRS group showed a significantly higher prevalence of hypertension, sleeplessness, and social isolation. The CRS group had a significantly higher SII-log value than the non-CRS group (Table 1).

**Table 1:** Characteristics of the dementia patients and control participants at baseline.

Characteristics	Overall (*N* = 364,945)	CRS cases (*N* = 7,176)	Non-CRS cases (*N* = 357,769)	*P*-value
Mean follow-up time (median [IQR])	12 (0)	12 (0)	12 (0)	0.64
Age (median [IQR])	60 (13)	61 (12)	60 (13)	<0.01
Sex (%)				<0.01
Female	191,347 (52.4%)	4,166 (58.1%)	187,181 (52.3%)	
Male	173,598 (47.6%)	3,010 (41.9%)	170,588 (47.7%)	
Ethnicity				0.105
Not White	32,562 (8.9%)	601 (8.4%)	31,961 (8.9%)	
White	332,383 (91.1%)	6,575 (91.6%)	325,808 (91.1%)	
SII-log (median [IQR])	6.26 (0.60)	6.27 (0.62)	6.26 (0.60)	0.017
Education (%)				0.85
Lower	236,229 (64.7%)	4,709 (65.6%)	231,520 (64.7%)	
Higher	128,716 (35.3%)	2,467 (34.4%)	126,249 (35.3%)	
BMI				0.13
Normal weight	122,072 (33.4%)	2,316 (32.3%)	119,756 (33.5%)	
Underweight	1795 (0.5%)	38 (0.5%)	1757 (0.5%)	
Overweight	156,812 (43.0%)	3,103 (43.2%)	153,709 (43.0%)	
Obesity	84,266 (23.1%)	1719 (24.0%)	82,547 (23.1%)	
TDI (%)				0.002
Lower	73,457 (20.1%)	1,464 (20.4%)	71,993 (20.1%)	
Low	73,127 (20.0%)	1,515 (21.1%)	71,612 (20.0%)	
Mid	73,022 (20.0%)	1,470 (20.5%)	71,552 (20.0%)	
High	72,757 (19.9%)	1,427 (19.9%)	71,330 (19.9%)	
Higher	72,582 (19.9%)	1,300 (18.1%)	71,282 (19.9%)	
PRS-AD (%)				0.19
Low	72,968 (20.0%)	1,393 (19.4%)	71,575 (20.0%)	
Mid	218,943 (60.0%)	4,380 (61.0%)	214,563 (60.0%)	
High	73,034 (20.0%)	1,403 (19.6%)	71,631 (20.0%)	
Smoking status (%)				<0.01
Never	200,670 (55.0%)	3,945 (55.0%)	196,725 (55.0%)	
Previous	127,529 (34.9%)	2,649 (36.9%)	124,880 (34.9%)	
Current	36,746 (10.1%)	582 (8.1%)	36,164 (10.1%)	
Alcohol consumption (%)				0.51
Never/Previous	14,123 (3.9%)	289 (4.0%)	13,834 (3.9%)	
Current	350,822 (96.1%)	6,887 (96.0%)	343,935 (96.1%)	
Level of physical activity				0.008
Low	67,921 (18.6%)	1,432 (20.0%)	66,489 (18.6%)	
Moderate	149,029 (40.8%)	2,917 (40.6%)	146,112 (40.8%)	
High	147,995 (40.6%)	2,827 (39.4%)	145,168 (40.6%)	
Hypertension (%)	85,952 (23.6%)	1762 (24.6%)	84,190 (23.5%)	0.044
Cancer (%)	47,448 (13.0%)	955 (13.3%)	46,493 (13.0%)	0.47
Diabetes (%)	18,591 (5.1%)	346 (4.8%)	18,245 (5.1%)	0.30
Stroke (%)	514 (0.1%)	9 (0.1%)	505 (0.1%)	0.063
Sleeplessness (%)				<0.001
Never/Sometimes	265,721 (72.8%)	4,828 (67.3%)	260,893 (72.9%)	
Usually	99,224 (27.2%)	2,348 (32.7%)	96,876 (27.1%)	
Social isolation (%)	29,924 (8.2%)	525 (7.3%)	29,399 (8.2%)	0.006

*P*-value is based on the chi-squared test for categorical variables and the *t*-test for continuous variables.

### 3.2 Cohort analysis

**Figure 2:**
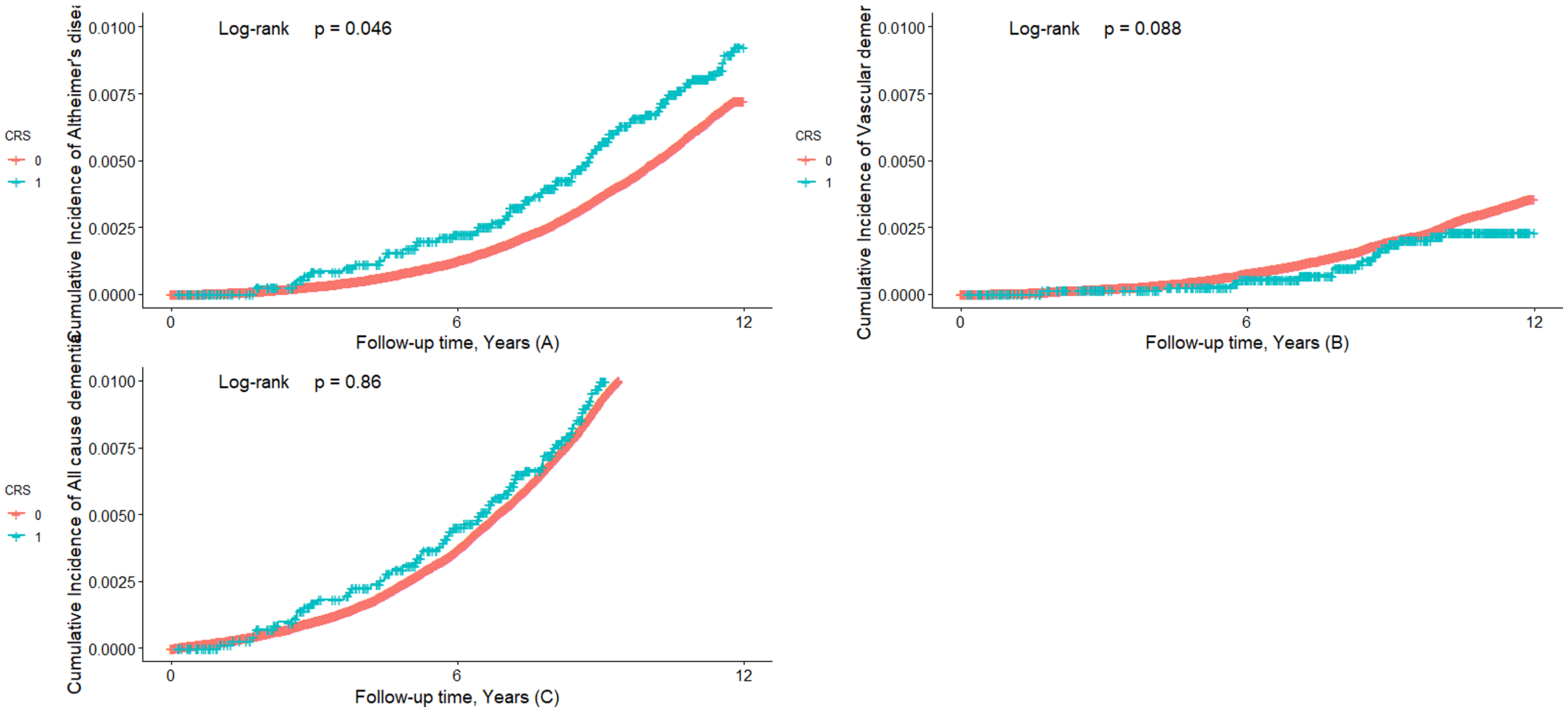
Survival analyses of dementia cumulative incidence. Kaplan–Meier survival analyses were performed to evaluate the cumulative incidence of dementia and its different sub-phenotypes. Figure **(A)** shows the cumulative incidence of Alzheimer’s disease between the CRS group and the non-CRS group. Figure **(B)** shows the cumulative incidence of vascular dementia between the CRS group and the non-CRS group. Figure **(C)** shows the cumulative incidence of all-cause dementia between the CRS group and the non-CRS group.

We investigated the association between CRS and the risk of all-cause dementia, AD, and VD by performing a Cox proportional hazards regression model. Compared to the participants without CRS, the CRS cases had a significantly higher risk of AD (HR: 1.33, 95%CI: 1.04–1.71, *p* = 0.023) (Table 2 and Figure 2A). However, the CRS cases did not have a significantly higher risk of all-cause dementia (HR: 1.04, 95%CI: 0.86–1.26, *p* = 0.67) and VD (HR: 0.65, 95%CI: 0.40–1.07, *p* = 0.091) (Table 2 and Figures 2B,C).

**Table 2:** Association of CRS with all-cause dementia, Alzheimer’s disease, and vascular dementia.

Types of dementia	Model 1		Model 2		Model 3	
	HR [95% CI]	*P*-value	HR [95% CI]	*P*-value	HR [95% CI]	*P*-value
Alzheimer’s disease	1.29(1.003–1.65)	0.047*	1.31(1.02–1.68)	0.033*	1.33(1.04–1.71)	0.023*
All-cause dementia	1.02(0.84–1.23)	0.86	1.03(0.85–1.25)	0.75	1.04(0.86–1.26)	0.67
Vascular dementia	0.69(0.42–1.12)	0.13	0.68(0.41–1.11)	0.12	0.65(0.40–1.07)	0.091

We obtained the HR and 95%CI using Cox regression.

Model 1 was a crude model without adjustment for covariates.

Model 2 was adjusted for age, sex, ethnicity, BMI, Townsend deprivation index, and standardized PRS for Alzheimer’s disease.

Model 3, based on Model 2, was additionally adjusted for education level, alcohol consumption, smoking status, physical activity level, social isolation status, sleeplessness, hypertension, diabetes, stroke, and cancer.

BMI, body mass index; CI, confidence interval; HR, hazard ratio.

*p < 0.05; **p < 0.01.

### 3.3 The mediation effect of inflammation markers

**Figure 3:**
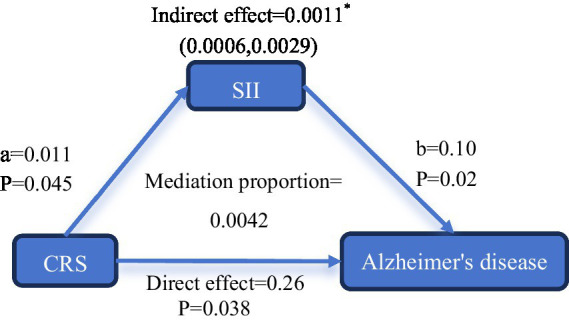
Mediation analysis path diagram. Mediation analysis of the SII on the association between CRS and Alzheimer’s disease. Mediation proportion = Indirect effect/[Indirect effect + Direct effect]. The analysis was adjusted for age, sex, ethnicity, education, Townsend deprivation index, alcohol consumption, smoking status, BMI, physical activity level, social isolation status, sleeplessness, hypertension, diabetes, stroke, cancer, and standardized PRS for Alzheimer’s disease. *p < 0.05, **p < 0.001, ***p < 0.0001.

In the mediation analysis, only the SII showed a significant mediating role between CRS and AD. The direct effect between CRS and AD was 0.26 (95%CI: 0.01–0.51, *p* = 0.038). The estimated value of the impact of CRS on the SII (a) was 0.011 (95%CI: 0.0002–0.021, *p* = 0.045), and the estimated value of the impact of the SII on the outcome (b) was 0.10 (95%CI: 0.016–0.18, *p* = 0.02). Therefore, the indirect effect (a*b) was 0.0011 (95%CI: 0.0006–0.0029), indicating that the increased inflammation level due to CRS may contribute to AD. In addition, the mediation proportion results showed that inflammation status could mediate the association between CRS and AD, and the SII level positively explained the association between CRS and AD, with a value of 0.0042 (Figure 3). The mediation results of all-cause dementia and VD were not significant and are shown in Supplementary materials 6, 7.

### 3.4 Subgroup analysis and sensitivity analysis

**Figure 4:**
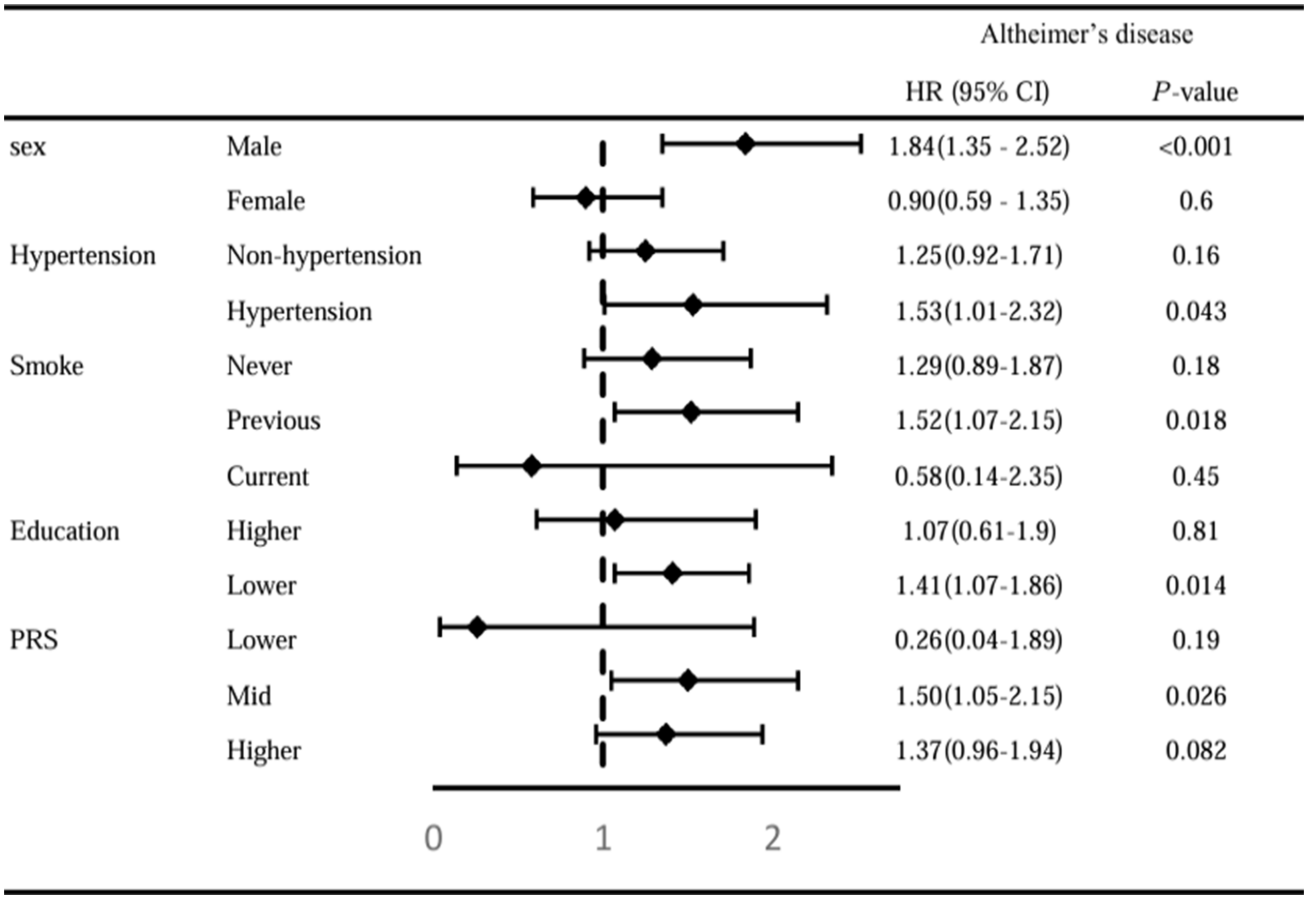
Stratified analysis by sex, hypertension, smoking status, education level, and PRS-AD.

To identify the influence of important variables on the association between CRS and dementia disease, we performed a series of subgroup analyses by sex, education level, hypertension, PRS-AD, and smoking status. In the sex subgroup analysis, CRS was associated with a higher risk of all-cause dementia (HR: 1.37, 95%CI: 1.08–1.73, *p* = 0.01) and AD (HR: 1.84, 95%CI: 1.35–2.52, *p* < 0.001) only in the male participants. In the hypertension subgroup analysis, CRS was associated with a higher risk of AD (HR: 1.53, 95%CI: 1.01–2.32, *p* = 0.043) in the hypertension group. In the smoking status-stratified analysis, CRS was significantly associated with a higher risk of AD (HR: 1.52, 95%CI: 1.07–2.15, *p* = 0.018) only in the former smokers group. In the education subgroup analysis, we divided the participants into two groups according to education level: college or above and below college. CRS was significantly associated with a higher risk of AD (HR: 1.41, 95%CI: 1.07–1.86, *p* = 0.014) in the low education group only. In PRS-AD subgroup analysis, CRS was significantly associated with a higher risk of AD (HR: 1.50, 95%CI: 1.05–2.15, *p* = 0.026) in the mid PRS-AD group only (Figure 4 and Supplementary material 8).

In the sensitivity analysis, the results of the competing risk models were in accordance with the results of the main Cox regression model (Table 3). After excluding participants with ≤2 years of follow-up, CRS was still significantly associated with a higher risk of AD (Table 4). After excluding self-reported CRS cases, the results were consistent with the results of the Cox regression model, and the HR estimates were higher than those from the main Cox regression model (Table 5).

**Table 3:** Sensitivity analysis 1—death competing risk model.

Types of dementia	Model 1		Model 2		Model 3	
	HR [95% CI]	*P*-value	HR [95% CI]	*P*-value	HR [95% CI]	*P*-value
Alzheimer’s disease	1.29(1.01–1.66)	<0.05*	1.33(1.04–1.70)	<0.05*	1.35(1.05–1.73)	<0.05*
All-cause dementia	1.05(0.87–1.28)	>0.05	1.04(0.86–1.26)	>0.05	1.02(0.84–1.24)	>0.05
Vascular dementia	0.70(0.42–1.14)	>0.05	0.67(0.41–1.1)	>0.05	0.66(0.40–1.07)	>0.05

We obtained the HR and 95%CI using the death competing risk model.

Model 1 was a crude model without adjustment for covariates.

Model 2 was adjusted for age, sex, ethnicity, BMI, Townsend deprivation index, and standardized PRS for Alzheimer’s disease.

Model 3, based on Model 2, was additionally adjusted for education level, alcohol consumption, smoking status, physical activity level, social isolation status, sleeplessness, hypertension, diabetes, stroke, and cancer.

BMI, body mass index; CI, confidence interval; HR, hazard ratio.

*p < 0.05; **p < 0.01.

**Table 4:** Sensitivity analysis 2—excluding participants with ≤2 years of follow-up time.

Types of dementia	Model 1		Model 2		Model 3	
	HR [95% CI]	*P*-value	HR [95% CI]	*P*-value	HR [95% CI]	*P*-value
Alzheimer’s disease	1.25(0.97–1.62)	0.087	1.27(0.99–1.65)	0.065	1.30(1.0–1.68)	0.047*
All-cause dementia	1.01(0.83–1.23)	0.94	1.02(0.84–1.24)	0.83	1.04(0.85–1.27)	0.70
Vascular dementia	0.69(0.42–1.12)	0.13	0.67(0.41–1.1)	0.12	0.65(0.4–1.07)	0.091

We obtained the HR and 95%CI using Cox regression.

Model 1 was a crude model without adjustment for covariates.

Model 2 was adjusted for age, sex, ethnicity, BMI, Townsend deprivation index, and standardized PRS for Alzheimer’s disease.

Model 3, based on Model 2, was additionally adjusted for education level, alcohol consumption, smoking status, physical activity level, social isolation status, sleeplessness, hypertension, diabetes, stroke, and cancer.

BMI, body mass index; CI, confidence interval; HR, hazard ratio.

*p < 0.05; **p < 0.01.

**Table 5:** Sensitivity analysis 3—excluding self-reported CRS cases.

Types of dementia	Model 1		Model 2		Model 3	
	HR [95% CI]	*P*-value	HR [95% CI]	*P*-value	HR [95% CI]	*P*-value
Alzheimer’s disease	1.52 (1.14–2.04)	<0.01*	1.50 (1.12–2.01)	<0.01*	1.51 (1.13–2.02)	<0.01*
All-cause dementia	1.24 (0.99–1.54)	0.061	1.22 (0.98–1.52)	0.082	1.21 (0.97–1.51)	0.092
Vascular dementia	0.87 (0.51–1.51)	0.63	0.87 (0.50–1.5)	0.62	0.87(0.5–1.5)	0.61

We obtained the HR and 95%CI using Cox regression.

Model 1 was a crude model without adjustment for covariates.

Model 2 was adjusted for age, sex, ethnicity, BMI, Townsend deprivation index, and standardized PRS for Alzheimer’s disease.

Model 3, based on Model 2, was additionally adjusted for education level, alcohol consumption, smoking status, physical activity level, social isolation status, sleeplessness, hypertension, diabetes, stroke, and cancer.

BMI, body mass index; CI, confidence interval; HR, hazard ratio.

*p < 0.05; **p < 0.01.

## 4 Discussion

We conducted this cohort study to explore the association between CRS and dementia. The results of the Cox regression model showed that CRS was significantly associated with a higher risk of AD but not associated with the risk of all-cause dementia and VD. In addition, we found that this relationship between CRS and Alzheimer’s disease could be mediated by the SII, with a mediation proportion of 0.0042. In the stratified analysis, the male participants, participants with hypertension, former smokers, participants with less than a college education, and participants with a medium PRS-AD were more susceptible to AD. The male participants and participants with hypertension were more susceptible to all-cause dementia. According to our results, CRS might be a risk factor for dementia, and inflammation caused by CRS may mediate the relationship between CRS and AD.

Previous cross-sectional studies (Jung et al., 2021; Chung et al., 2015) have reported that CRS is positively associated with dementia, which is consistent with our findings. However, our results differ from those of a previous longitudinal study (Son et al., 2024), which showed that CRS was not associated with dementia and its subtypes. Compared to this longitudinal study, our cohort was larger, had a longer follow-up period of up to 12 years, and included a broader age range. In addition, the UK Biobank primarily included participants of European ancestry, which differs from the Korean participants of Asian ancestry in terms of genetic background and body composition. In summary, compared to previous studies, our study involved a larger population, a longer follow-up period, and the use of mediation analysis to investigate the association between CRS and dementia.

The association between CRS and dementia differed in the sex subgroup analysis. Apart from the ICD-10 classification criteria, CRS can be divided into three main types: CRS with nasal polyps, CRS without nasal polyps, and allergic fungal rhinosinusitis. Among these three types, CRS with nasal polyps is considered the most severe and is more difficult to treat or control due to the high likelihood of polyp recurrence (Mullol et al., 2022). In addition, a study reported that CRS with nasal polyps has a higher prevalence in male individuals (Ramkumar et al., 2023). Overall, due to the recurrence of nasal polyps, the difficulty in treating CRS with nasal polyps, and its higher prevalence in male individuals, the association between CRS and dementia may be more easily observed in male individuals. Participants with lower education levels may be associated with lower socioeconomic status (Lai et al., 2023) or may have limited time for treatment, which may make them more prone to developing dementia.

Although the etiology of CRS and dementia is not yet fully understood, several hypotheses regarding the mechanisms of dementia have been proposed, such as the hyperphosphorylated tau protein and amyloid-β hypothesis (Ashrafian et al., 2021; Zhang et al., 2021), the oxidative stress hypothesis (Bai et al., 2022), and the inflammation hypothesis (Guerrero et al., 2021; Irwin and Vitiello, 2019). The CRS may increase the risk of Alzheimer’s disease through chronic inflammation. The blood–brain barrier could be disrupted in the progression of neurodegenerative diseases, which might make the brain susceptible to inflammation status (Sweeney et al., 2018). The sinuses are close to the brain in physical distance, and there are several kinds of opportunistic pathogens in the sinuses, which can potentially cause neuroinflammation and aggregate CRS when the body’s conditions allow (Lal et al., 2017). Neuroinflammation could affect different kinds of microglia and ultimately facilitate the progression of Alzheimer’s disease (Kwon and Koh, 2020; Leng and Edison, 2021). In addition, neutrophils may play a significant role and proliferate during CRS (Delemarre et al., 2021). Neutrophils could produce platelet-activating factors to aggregate and increase the level of platelets (Gill et al., 2015), which could increase the level of the SII. However, according to a review in 2023 (Xie et al., 2023), inflammation caused by CRS can be divided into three types, all characterized by abnormal levels of special cell cytokines. As an important type of white blood cell with the largest quantity, neutrophils may cause a cascade effect of inflammation, leading to a significant increase in cell cytokines (Megha et al., 2021). Therefore, the SII may not be the main path from CRS to dementia, and cell cytokines may play a key role, which means that the effect of cell cytokines should be explored carefully in future studies.

Apart from inflammation, other symptoms caused by CRS, such as loss of smell and infections, have also been associated with dementia. A cohort study showed that olfactory dysfunction may be a risk factor for amnestic mild cognitive impairment and AD (Roberts et al., 2016). According to previous studies, inflammation caused by CRS with nasal polyposis could contribute to olfactory dysfunction and cause the volume of the olfactory bulb to decrease (Huang et al., 2024; Shehata et al., 2018). On the other hand, CRS could cause upper respiratory infections (Volpe et al., 2023), which may, in turn, induce the appearance of inflammation and ultimately contribute to olfactory dysfunction (Shehata et al., 2018). In the brain, the entorhinal cortex is close to the olfactory system and plays a significant role in attention, conditioning, event processing, and spatial cognition (Coutureau and Di Scala, 2009), etc. In the preclinical stage of AD, specific dysfunction of the entorhinal cortex can be observed (Igarashi, 2023). This impairment might lead to hyperactivation of adjacent brain areas (such as the hippocampus and olfactory bulb), potentially resulting in hippocampus and olfactory bulb degeneration (Dan et al., 2021). In addition, reduced oxygen uptake due to nasal congestion caused by CRS may contribute to dementia (Li et al., 2018; Liu et al., 2023).

Overall, the underlying mechanism of this relationship needs further exploration. From a public health perspective, understanding this relationship could help control dementia better. From a clinical perspective, understanding this relationship could help prevent AD by reducing or eliminating inflammation status caused by CRS. In life, people should take CRS seriously and seek active treatment as soon as they are diagnosed. According to the mediation analysis results, inflammation status only accounts for a small portion of the risk from CRS to AD. Therefore, exploring the underlying relationship between CRS and dementia and identifying other mediation factors through clinical approaches is important for treating patients with CRS. In addition, investigating the relationship between CRS and dementia can help us more comprehensively understand the risks associated with CRS and motivate healthcare workers to find better ways to treat patients with CRS.

Our study has several strengths. First, it was a prospective study, which provided a fixed sequence of time from exposure to outcome. Therefore, it could offer more reliable evidence compared to cross-sectional studies. Second, compared to previous studies, we conducted a cohort study with a longer follow-up period and a larger population, and we performed three sensitivity analyses to evaluate the robustness of our results. Third, we were the first to investigate the effect of inflammation status caused by CRS on the development of dementia through mediation analysis, which provided evidence for the role of inflammation in the process of dementia. Fourth, we initially investigated the mediation role of the inflammation index in the association between CRS and AD, providing new insights into the underlying mechanisms of CRS.

However, our study also has some limitations. First, the majority of participants in the UKB were White people of European ancestry, which may limit the generalizability of our results. Second, the inflammation mediation analysis was limited to blood cell counts and derived ratios, without including subsets of inflammatory cells or cytokines produced by inflammatory cells. Third, reverse causation cannot be completely ruled out because cohort studies cannot avoid reverse causation. Fourth, due to information unavailability, we could not classify CRS into CRS with nasal polyps and CRS without nasal polyps. Since different types of CRS might have different effects on AD, we could only explore the overall effect of CRS on AD, and this might increase the gap between data research and clinical applications. Fifth, because we excluded more than 1,30,000 participants, the statistical power may be decreased and the interpretation of the results requires caution. Sixth, the mediation proportion was very small, suggesting that its actual impact may be limited. Therefore, the mechanism linking CRS and dementia requires further investigation. Finally, although our analysis was adjusted for several confounding factors, there were still some potential confounders that we could not take into consideration, such as the use of anti-inflammatory medications.

## 5 Conclusion

CRS may be associated with a higher risk of AD, and the association is mediated, in a very small part, by the SII. Our findings may provide some clues for research into the cause of AD.

## Data Availability

The datasets presented in this study can be found in online repositories. The names of the repository/repositories and accession number(s) can be found in the article/Supplementary material.
