# Supplementary material for: The association between chronic rhinosinusitis and the risk of dementia: a longitudinal study
Source: Front Aging Neurosci. 2025 Oct 6;17:1609790. doi: 10.3389/fnagi.2025.1609790 (PMC12535962; doi:10.3389/fnagi.2025.1609790)
Supplement: Supplementary file 1 [file Table_1.DOCX]

Supplement 1: the diagnose criteria of dementia

For all cause dementia, the UKB use ICD-09 codes 290, 291, 294, 331.0, 331 and ICD-10 codes A81.0, F00, F01, F02, F03, F05, F10, G30, G3, I67 to diagnose all cause dementia. For Alzheimer's disease, the UKB use ICD-09 codes 331.0 and ICD-10 codes F00.0, F00.1, F00.2, F00.9, G30 to diagnose Alzheimer's disease. For Vascular dementia, the UKB use ICD-09 290.4 and ICD-10 codes F01, F01.0, F01.1, F01.2, F01.3, I67 to diagnose Vascular dementia.

Supplement 2: the number of dementia cases

| Dementia | Number (percentage %) |
| --- | --- |
| All cause dementia | 5329(100) |
| Altheimer’s disease | 2538 (47.62) |
| Vascular dementia | 1232 (23.11) |
| Frontotemporal dementia | 168 (3.15) |
| Other unclassified dementia kinds | 1391 (26.12) |

| Covariates | Field number | Classification |
| --- | --- | --- |
| Age | 34 | Continuous |
| Sex | 31 | Female  male |
| Ethnic | 21000 | White  No White |
| Education | 6138 | College or above  Under the college |
| Townsend deprivation index (TDI) | 22189 | Divide evenly into five portions: Q1/Q2/Q3/Q4/Q5 |
| Smoke status | 20116 | Current  Previous  Never |
| Alcohol drinker status | 20117 | Current  Previous  Never |
| Body mass index (BMI) | 21001 | Lose weight: <18.5  Normal weight: 18.5~25  Overweight: 25~30  Obesity: >30 |
| Social isolation status | 1031 | Yes: participants with frequency of friend/family visits less than about once a month  No: participants with frequency of friend/family visits >=once a month |
| Sleepless | 1200 | Never or sometime  Usually |
| Hypertension | 6150&41270 | Yes/NO |
| Diabetes | 41270 | Yes/NO |
| Stroke | 41270 | Yes/NO |
| PRS-AD | 26206 | Low: <20th per  Mid: 20th per~80th per  High: >80th per |
| Physical activity level (according to International Physical Activity Questionnaire) | 22032 | High  Mid  Low |

Supplement 3: details of covariates

Supplement 4: the distribution of SII


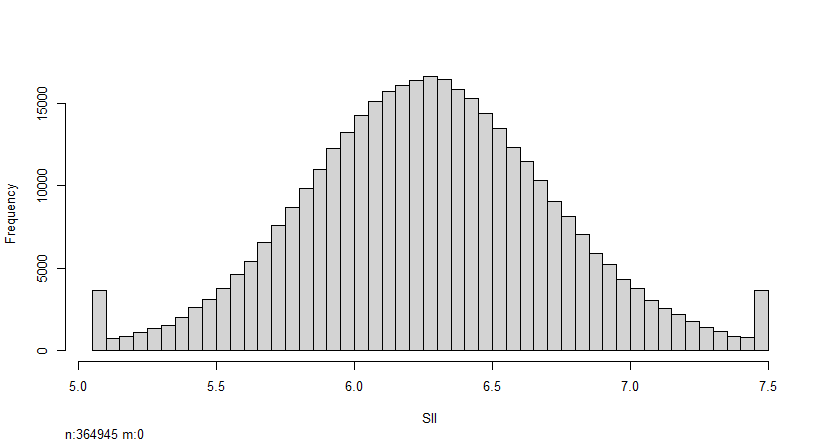


Supplement 5: The results of proportion hazard consumption. The P-values of the global Schoenfeld test results and CRS are 0.098 and 0.13, which can be considered this Cox regression model do not violate the proportion hazard consumption.


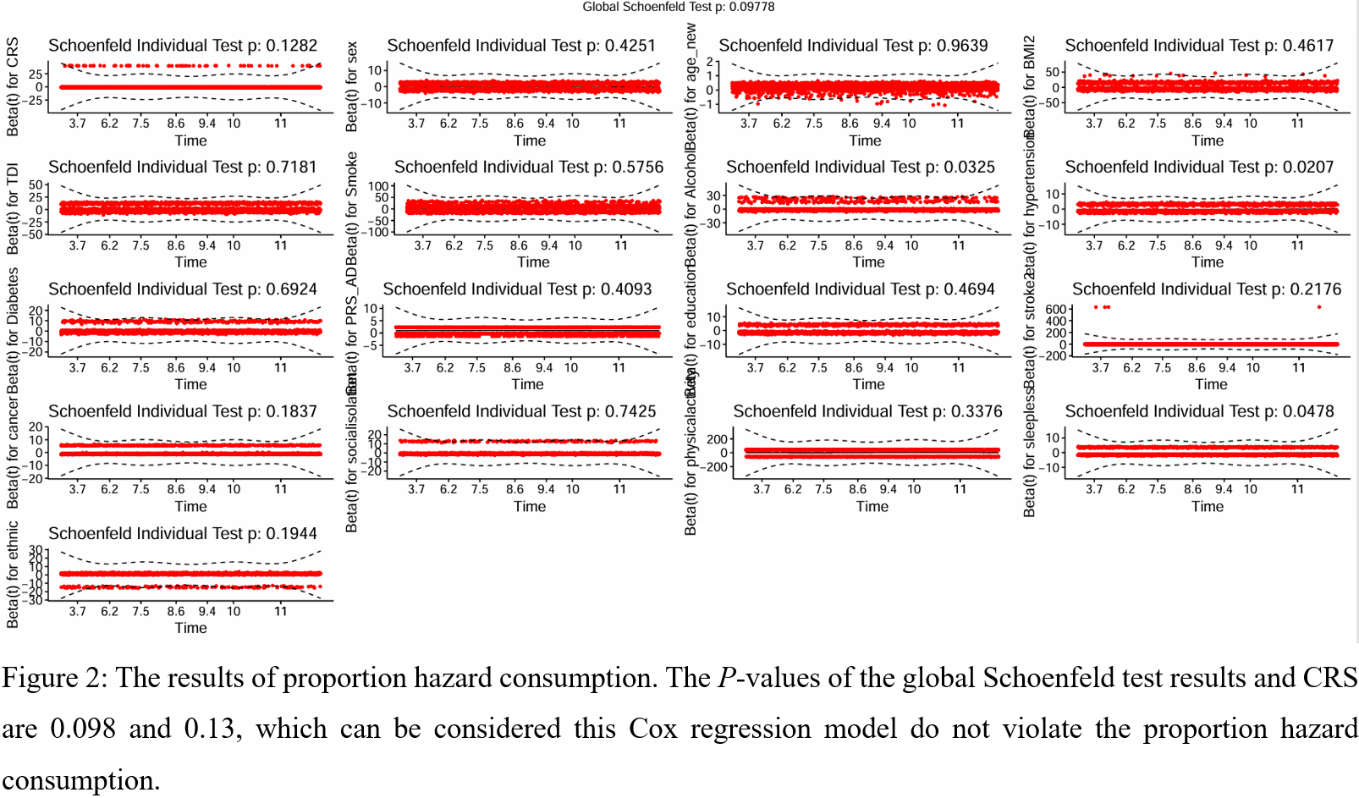


Supplement 6:

CRS

SII

ALL cause dementia

a= 0.012

P=0.029

b=0.17

P<0.001

Direct effect= 0.0469

P=0.64

Indirect effect=0.002^*^

(0.0002-0.004)

Mediation proportion=

0.042

Note: Mediation analysis path diagram. Mediation analysis of SII (systemic immune-inflammation index) on the association between CRS and all cause dementia. Mediation proportion= Indirect effect/ [Indirect effect+ Direct effect]. Adjusted for age / sex / ethnic / education / Townsend deprivation index / alcohol status / smoke status / BMI / physical activity level / social isolation status / sleepless / hypertension / diabetes / stroke / cancer and standard PRS for Alzheimer’s disease. *p < 0.05.

Supplement 7:

CRS

SII

Vascular dementia

a= 0.012

P=0.029

b=0.18

P=0.003

Direct effect= -0.36

P=0.15

Indirect effect=0.0021^*^

(0.0001,0.0049)

Mediation proportion=

0.0059

Note: Mediation analysis path diagram. Mediation analysis of SII (systemic immune-inflammation index) on the association between CRS and vascular dementia. Mediation proportion= Indirect effect/ [Indirect effect+ Direct effect]. Adjusted for age / sex / ethnic / education / Townsend deprivation index / alcohol status / smoke status / BMI / physical activity level / social isolation status / sleepless / hypertension / diabetes / stroke / cancer and standard PRS for Alzheimer’s disease. *p < 0.05.

Supplement 8: Stratified analysis by sex, hypertension, smoke status, education and PRS-AD

|  |  | All cause dementia | | Vascular dementia | |
| --- | --- | --- | --- | --- | --- |
|  |  | HR (95% CI) | *P*-value | HR (95% CI) | *P*-value |
| sex |  |  |  |  |  |
|  | Male | 1.37(1.08 - 1.73) | 0.01 | 1.0(0.57 - 1.72) | 0.99 |
|  | Female | 0.73(0.53 - 1.01) | 0.061 | 0.39(0.12-1.2) | 0.09 |
| Hypertension |  |  |  |  |  |
|  | Non-hypertension | 0.93(0.73 - 1.2) | 0.59 | 0.50(0.24 - 1.06) | 0.07 |
|  | Hypertension | 1.3(0.96 - 1.76) | 0.09 | 1.03(0.53 - 1.98) | 0.94 |
| Smoke |  |  |  |  |  |
|  | Never | 1.11(0.84 - 1.46) | 0.46 | 0.95(0.49 - 1.85) | 0.89 |
|  | Previous | 0.99(0.74 - 1.33) | 0.96 | 0.46(0.19 - 1.1) | 0.08 |
|  | Current | 1.11(0.57 - 2.15) | 0.76 | 0.86(0.21 - 3.47) | 0.83 |
| Education |  |  |  |  |  |
|  | Higher | 0.86(0.56 - 1.32) | 0.48 | 0.68(0.22 - 2.13) | 0.51 |
|  | Lower | 1.11(0.9 - 1.37) | 0.34 | 0.71(0.41 - 1.22) | 0.21 |
| PRS |  |  |  |  |  |
|  | Lower | 0.67(0.32 - 1.42) | 0.30 | 0.74(0.18 - 2.99) | 0.67 |
|  | Mid | 1.11(0.85 - 1.45) | 0.43 | 0.67(0.33 - 1.34) | 0.26 |
|  | Higher | 1.08(0.8 - 1.45) | 0.61 | 0.74(0.33 - 1.66) | 0.46 |
